# The prognostic significance of tyrosine-protein phosphatase nonreceptor type 12 expression in nasopharyngeal carcinoma

**DOI:** 10.1007/s13277-015-3176-x

**Published:** 2015-02-09

**Authors:** Xin-Ke Zhang, Miao Xu, Jie-Wei Chen, Feng Zhou, Yi-Hong Ling, Chong-Mei Zhu, Jing-Ping Yun, Mu-Yan Cai, Rong-Zhen Luo

**Affiliations:** 1 State Key Laboratory of Oncology in South China, Collaborative Innovation Center for Cancer Medicine, Sun Yat-sen University Cancer Center, Guangzhou, China; 2 Department of Pathology, Sun Yat-sen University Cancer Center, No. 651, Dongfeng Road East, 510060 Guangzhou, China; 3 Department of Medical Affairs, Sun Yat-sen University Cancer Center, Guangzhou, China

**Keywords:** Tyrosine-protein phosphatase nonreceptor type 12 (PTPN12), Nasopharyngeal carcinoma (NPC), Prognosis

## Abstract

Tyrosine-protein phosphatase nonreceptor type 12 (PTPN12) has been proposed to predict prognosis of various human cancers. However, the clinicopathologic and prognostic significance of PTPN12 expression in NPC has not yet been elucidated. The objective of this study was to investigate the clinicopathological and prognostic implication of PTPN12 in nasopharyngeal carcinoma (NPC) patients. Protein expression levels of PTPN12 were explored by semiquantitative immunohistochemical staining on archival formalin-fixed, paraffin-embedded pathological specimens consisting of 203 NPCs, and 40 normal nasopharyngeal mucosa tissues. Receiver operating characteristic (ROC) curve analysis was employed to determine the cutoff score of PTPN12 expression in NPCs. The PTPN12 immunohistochemical staining results were then correlated with various clinicopathological features and patients’ prognosis using various statistical models. Our results showed that decreased expression of PTPN12 was more frequently observed in NPC tissues compared with the normal nasopharyngeal mucosa. Further correlation analyses indicated that the decreased expression of PTPN12 was significantly associated with tumor T classification, N classification, distant metastasis, and clinical stage in NPCs (*P* < 0.05). Univariate analysis showed a significant association between the decreased expression of PTPN12 and adverse overall survival and disease-free survival (*P* < 0.05). More importantly, multivariate analysis identified the PTPN12 expression in NPC as an independent prognostic factor. The decrease expression of PTPN12 might be important in conferring a more aggressive behavior in NPC. Thus, PTPN12 expression may be used as a novel independent prognostic biomarker for patients with NPC.

## Introduction

Nasopharyngeal carcinoma (NPC) is the most common cancer originating from the nasopharynx, which is prevalent in south-east Asia, especially in southern China, where it constitutes a significant health burden [1]. Most of NPC cells are undifferentiated or poorly differentiated with the following characteristics: fast growth, invading adjacent regions, and metastasizing to regional lymph nodes and/or distant organs. With the advancement of the diagnostic and treatment techniques, the local-regional control rate for NPC has improved significantly in the past few decades, but the incidence of metastasis has not decreased greatly. If metastasis occurs, the outcome is very poor [2]. Epstein–Barr virus (EBV) infection is believed to be the major factor of NPC [3]. This infection initiates a multistep process with morphological progression involving multiple genetic and epigenetic events [1, 4]. Identification of molecular and biological changes that occur during carcinogenesis and/or progression could facilitate investigation of the signal pathway of NPC and generate new prognostic markers to more accurately predict patients’ clinical outcome and contribute to individualize treatments for NPC patients. Therefore, it is urgently needed to develop new biomarkers for clinical diagnosis/prognosis and find novel effective therapies for NPC.

Tyrosine-protein phosphatase nonreceptor type 12 (PTPN12) is one of ubiquitously expressed cytosolic protein tyrosine phosphatases (PTPs) family with an amino (N)-terminal PTP domain and a carboxyl (C)-terminal region involved in protein–protein interactions [5]. PTPs play an important role in signal transduction and regulation in cellular physiology and cancer [6, 7]. These PTPs can also serve as antagonists to tyrosine kinase (TK) signaling, thereby playing a prominent role in tumor suppression [8, 9]. PTPN12 is a key regulator of integrin-mediated adhesion and migration of endothelial cells by dephosphorylating the cytoskeleton regulators Cas, paxillin, and Pyk2, such an activity likely explains the critical role of PTPN12 in vascular development and tumor formation [10]. Also, PTPN12 is required for secondary T cell responses, energy prevention, and autoimmunity induction [11]. Recent studies showed that PTPN12 inhibits growth, proliferation, tumorigenicity, and metastatic potential in triple-negative breast cancer [12] and colon cancer [13], and also found to be a prognostic biomarker for esophageal squamous cell carcinoma [14]. However, there are no relevant reports on the prognostic value of PTPN12 in NPCs. In this study, we investigated the expression status of PTPN12 protein in NPC and normal nasopharyngeal tissues by tissue-microarray-based immunohistochemistry.

## Materials and methods

### Ethics statement

The study was approved by the Institute Research Medical Ethics Committee of Sun Yat-sen University. No informed consent (written or verbal) was obtained for use of retrospective tissue samples from the patients within this study, most of whom were deceased, since this was not deemed necessary by the ethics committee, who waived the need for consent. All samples were anonymized.

### Patients and tissue specimens

In this study, 203 specimens of NPC were collected in Sun Yat-sen University Cancer Center and in Guangdong Provincial People’s Hospital, Guangzhou, China, between January 1991 and August 2000. The selection of cases was based on the following criteria: pathologically confirmed as nonkeratinizing carcinoma of nasopharynx (World Health Organization types of II or III) with available biopsy specimens for tissue microarray (TMA) construction; no previous malignant disease or a second primary tumor; without radiotherapy, chemotherapy history before biopsy; Karnofsky ≥70; received radiotherapy (RT), induction chemotherapy/radiotherapy (IC/RT), or induction chemotherapy/chemoradiotherapy (IC/CRT) regimen; and follow-up regularly. Patients with unavailable biopsy tissues for constructing TMA were excluded from our study to provide adequate samples for pathological diagnosis. We chose 203 primary NPCs from the Department of Pathology of our institutes, and 40 samples of normal nasopharyngeal mucosa were used for controls. The routine staging workup was composed of a detailed physical examination, including fiber optic nasopharyngoscopy, magnetic resonance imaging (MRI) of the entire neck, chest X-ray, abdominal ultrasonography, a complete blood count, and a biochemical profile. The clinical stage was defined on the basis of the International Union Against Cancer (UICC) and the American Joint Committee on Cancer (AJCC) [15]. The Institute Research Medical Ethics Committee of Sun Yat-Sen University Cancer Center granted approval for this study.

### Follow-up

The response of RT or CRT was assessed clinically for primary lesion based on fiber optic nasopharyngoscopy and MRI 1 month after treatment. In our study, the patients were followed every 3 months for the first year and then every 6 months for the next 2 years and finally annually, thereafter. The diagnostic examinations consisted of fiber optic nasopharyngoscopy, MRI, CT, chest X-ray, abdominal ultrasonography, and bone scan when necessary to detect recurrence and/or metastasis. The total follow-up period was defined as the time from diagnosis to the date of death or the last date censored if patients were still alive.

### Tissue microarray

TMA was constructed in accordance with a previously described method [16]. In brief, the paraffin-embedded tissue blocks and the corresponding histological H&E-stained slides were overlaid for tissue TMA sampling. Duplicate of 0.6 mm diameter cylinders were punched from representative tumor areas of individual donor tissue block, and re-embedded into a recipient paraffin block at a defined position, using a tissue arraying instrument (Beecher Instruments, Silver Spring, MD, USA).

### Immunohistochemistry

Formalin-fixed, paraffin-embedded NPC samples were cut into 4-μm thick sequential sections and processed for immunohistochemistry (IHC) according to the previously described protocol [17]. The TMA slides were deparaffinized in xylene, rehydrated through graded alcohol, immersed in 3 % hydrogen peroxide for 10 min to block endogenous peroxidase activity and antigen retrieved by pressure cooking for 3 min in citrate buffer (pH = 6). For blocking nonspecific binding, the slides were preincubated with 10 % normal goat serum at room temperature for 20 min. Subsequently, the slides were incubated with rabbit polyclonal antibody anti-PTPN12 (1:300 dilution), overnight at 4 °C in a moist chamber. The slides were sequentially incubated with a secondary antibody (Envision, Dako, Denmark) for 30 min in the incubator at 37 °C and stained with 3,3-diaminobenzidine (DAB). Finally, the sections were counterstained with Mayer’s hematoxylin, dehydrated, and mounted. A negative control was obtained by replacing the primary antibody with a normal rabbit IgG.

### IHC evaluation

According to our previous study [17], protein expression levels of PTPN12 were evaluated by microscopic examination of stained TMA slides. The presence of cytoplasmic brown granules was considered to be positive for PTPN12 expression. In brief, the expression pattern was assessed as follows: each TMA spot was assigned an intensity score from 0 to 3 (I0; I1; I2; I3, 0, negative; 1, weak; 2, moderate; and 3, strong). Then, cytoplasmic PTPN12 was evaluated according to the percentage of positively stained cells in 5 % increments from 0 to 100 %. The final H score (range, 0–300) was determined by adding the sum of the scores obtained for each intensity and the proportion of the area stained (H score = I1*P1 + I2*P2 + I3*P3). PTPN12 expression was assessed by two independent pathologists (Zhang and Luo) who were blinded to the clinicopathological data. All lab methods were used for both tumor and nontumor specimens.

### Selection of cutoff score

The plot of sensitivity versus 1-specificity across varying cutoffs generates a curve in the unit square called an receiver operating characteristic (ROC) curve, the optimal cutoff value can be determined using ROC curve analysis by the point (0.0, 1.0) or (1.0, 0.0) [18, 19], at the PTPN12 score, the sensitivity and specificity for each outcome under study was plotted, and generating various ROC curves. The score was selected as the cutoff value which was closest to the point with both maximum sensitivity and specificity. The score below or equal to the cutoff value was served as decreased expression of PTPN12, on the contrary, above the cutoff value was seen as normal expression. To use ROC curve analysis, the clinicopathological characteristics were involved: T classification (T1–T2 versus T3–T4), N classification (N0–N1 versus N2–N3), distant metastasis (M0 versus M1), clinical stage (I–II versus III–IV), survival status (death due to NPC versus censored), and cancer recurrence (yes versus no).

### Statistical analysis

Statistical analyses were performed using SPSS software, version 16.0 (SPSS, Chicago, USA). The correlation between PTPN12 expression and clinicopathological features of NPC patients was assessed by Chi-square test. Univariate analysis of overall survival (OS; the proportion of cancer patients who survived for a specified time interval after diagnosis) and disease-free-survival (DFS) data were performed using the Kaplan–Meier method. The Cox proportional hazards regression model was used to identify the independent prognostic factors. A two-tailed *P* value of less than 0.05 was considered as statistically significant in all cases.

## Results

### Patients’ characteristics

The clinicopathological characteristics of NPC patients were detailed in Table 1. This NPC cohort consisted of 148 (72.9 %) men and 55 (27.1 %) women with median age of 47 years. Average follow-up period was 72.9 months (median, 73.0 months; range, 3.0 to 233.0 months); 138 patients (68.0 %) were diagnosed at late stages (III and IV), and the other 65 patients (32.0 %) were at early stages (I and II).

**Table 1:** Correlation between the expression of PTPN12 and clinicopathological features in nasopharyngeal carcinomas

	All cases	PTPN12 protein		
		Decreased expression	Normal expression	*P* value^a^
Sex				
Female	55	38 (69.1 %)	17 (30.9 %)	0.180
Male	148	87 (58.8 %)	61 (41.2 %)	
Age at diagnosis (years)				
≤45	86	55 (64.0 %)	31 (36.0 %)	0.551
>45	117	70 (59.8 %)	47 (40.2 %)	
Histological classification (WHO)				
Type II	53	31 (58.5 %)	22 (41.5 %)	0.591
Type III	150	94 (62.7 %)	56 (37.3 %)	
T classification				
1	26	13 (50.0 %)	13 (50.0 %)	0.010
2	67	40 (59.7 %)	27 (40.3 %)	
3	65	35 (53.8 %)	30 (46.2 %)	
4	45	37 (82.2 %)	8 (17.8 %)	
N classification				
0	40	18 (45.0 %)	22 (55.0 %)	0.000
1	94	49 (52.1 %)	45 (47.9 %)	
2	48	41 (85.4 %)	7 (14.6 %)	
3	21	17 (81.0 %)	4 (19.0 %)	
Distant metastasis				
0	152	87 (57.2 %)	65 (42.8 %)	0.028
1	51	38 (74.5 %)	13 (25.5 %)	
Clinical stage				
I	10	3 (30.0 %)	7 (70.0 %)	0.005
II	55	28 (50.9 %)	27 (49.1 %)	
III	78	48 (61.5 %)	30 (38.5 %)	
IV	60	46 (76.7 %)	14 (23.3 %)	

^a^Chi-square test; WHO, World Health Organization

### Selection of the cutoff score for PTPN12 expression

**Fig. 1:**
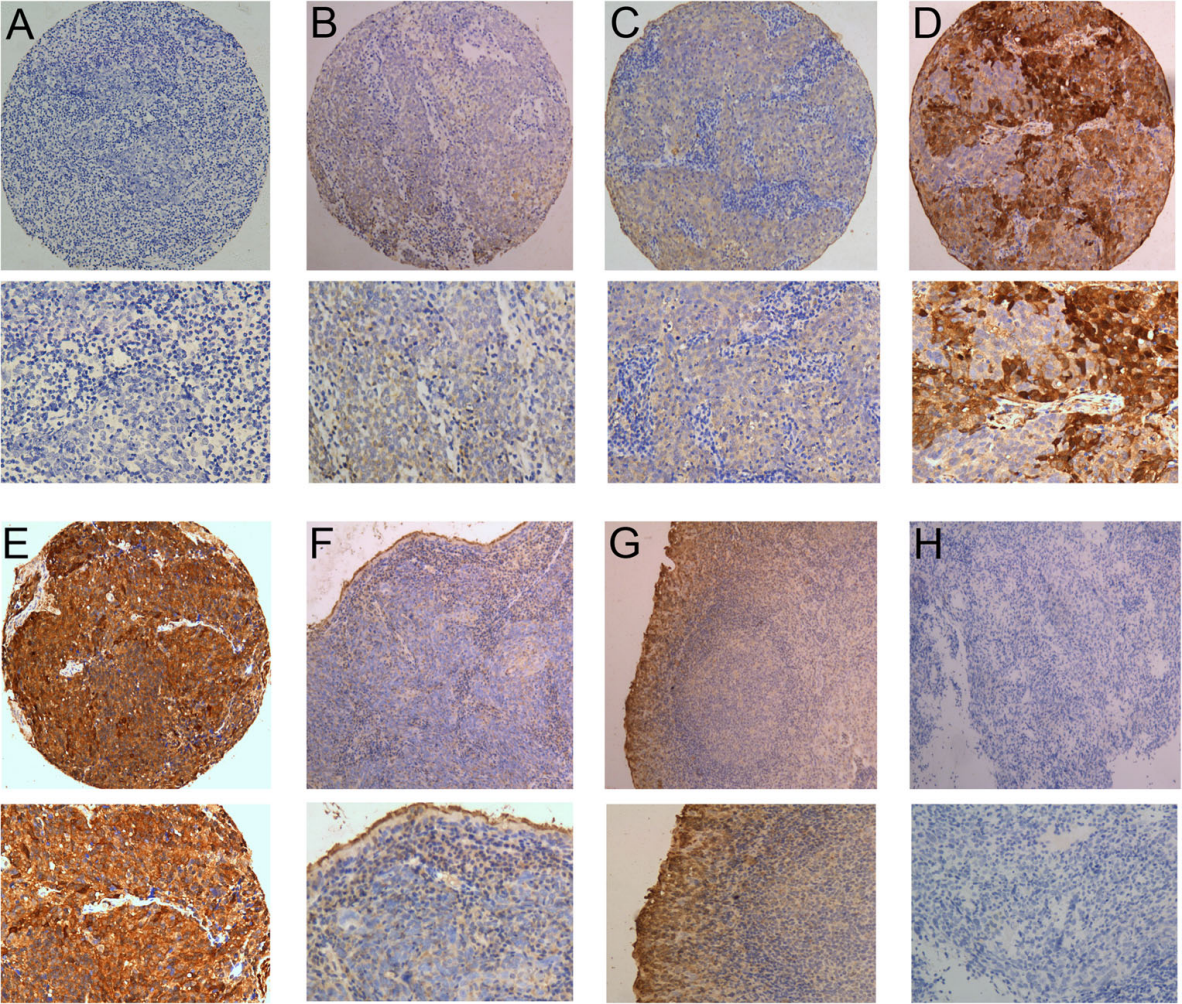
The expression pattern of PTPN12 protein in NPC and noncancerous nasopharyngeal tissues. **a** Negative expression of PTPN12 was shown in a NPC case (×100). **b** The micrograph with the H score <50 of PTPN12 IHC was shown in a NPC case (×100). **c** A NPC case demonstrated the expression of PTPN12 with the H score of 50 ~ 200 (×100). **d** A NPC case demonstrated the expression of PTPN12 with the H score of 225 (×100). **e** A NPC case demonstrated the expression of PTPN12 with the H score over 250 (×100). **f** NPC tissue demonstrated decreased expression of PTPN12 protein with the control of normal expression of PTPN12 in normal nasopharyngeal mucosal tissue (×100). **g** Normal expression of PTPN12 protein was shown in normal nasopharyngeal mucosal tissue (×100). **h** The negative control staining. The *lower panels* indicated the higher magnification (×200) from the *upper panels*

Since the IHC scores were evaluated semiquantitatively, in our study, we utilized ROC curve analysis to avoid the use of predetermined and often arbitrarily set cutoff values. ROC curves are commonly used in clinical oncology to evaluate and compare the sensitivity and specificity of diagnostic tests. Moreover, they allow one to identify the threshold value above which a test result should be considered positive for some outcome (Fig. 1). In immunohistochemical evaluation, the score with the shortest distance from the curve to the point with both maximum sensitivity and specificity, i.e., the point (1.0, 0.0) or (0.0, 1.0), was selected as the cutoff score leading to the greatest number of tumors correctly classified as having or not having the clinical outcome [19, 20].

**Fig. 2:**
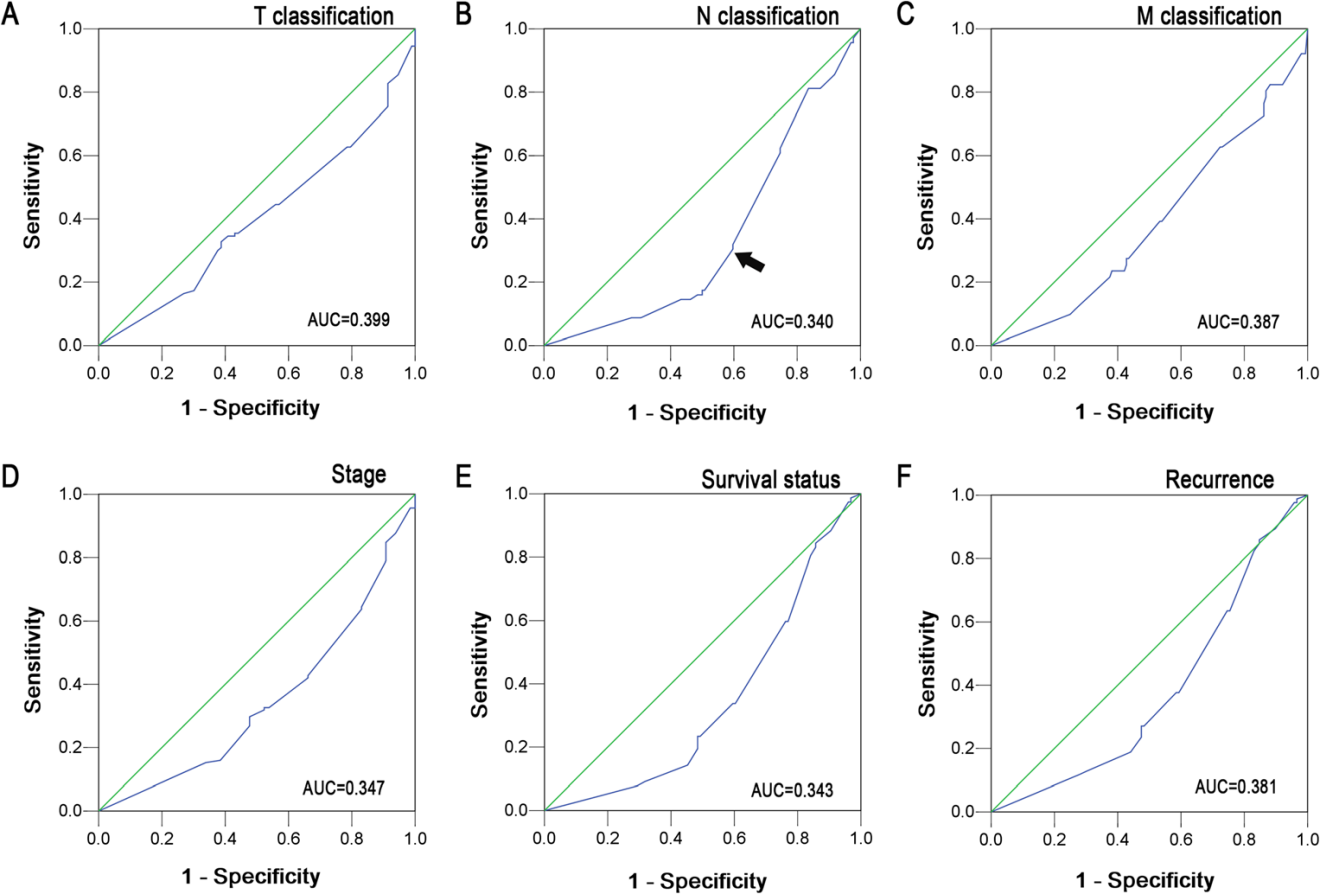
ROC curve analysis was conducted to determine the cutoff score for decreased PTPN12 expression. The sensitivity and specificity for each outcome were plotted: T classification (**a**), N classification (**b**), M classification (**c**), stage (**d**), survival status (**e**), and recurrence (**f**)

To select an optimal PTPN12 cutoff score for further analysis, the ROC curves for each clinicopathological feature (Fig. 2) show the arrow on the curve closest to the point (1.0, 0.0), which maximizes both the sensitivity and specificity for the outcome [17, 19], cancers with score above the obtained cutoff value were considered as normally expressed PTPN12, which led to the greatest number of cancers classified as having or not having the clinical outcome. As it was shown in Fig. 2, the N classification had the closest to the point (1.0, 0.0). Based on this outcome, we selected a PTPN12 expression score of 225 defined by the N classification as the optimal cutoff value for survival analysis. According to the ROC curve analysis, decreased expression of PTPN12 could be examined in 125/203 (61.6 %) of NPCs and in 14/40 (35.0 %) of normal nasopharyngeal mucosa, respectively. Decreased expression of PTPN12 in normal nasopharyngeal tissues is significantly lower than that in NPC (*P* < 0.001).

### The relationship between PTPN12 expression and the clinicopathological features of NPC patients

The rates of normal and decreased expression of PTPN12 in NPCs about several clinicopathological features were detailed in Table 1. The results showed that decreased expression of PTPN12 was significantly correlated with cancer T classification, N classification, distant metastasis, and clinical stage (*P* < 0.05; Table 1), and there was no significant association between PTPN12 expression and other clinicopathological features, such as patient sex, age, and cancer histological classification (*P* > 0.05; Table 1).

### The relationship between PTPN12 expression and NPC patients’ survival

**Fig. 3:**
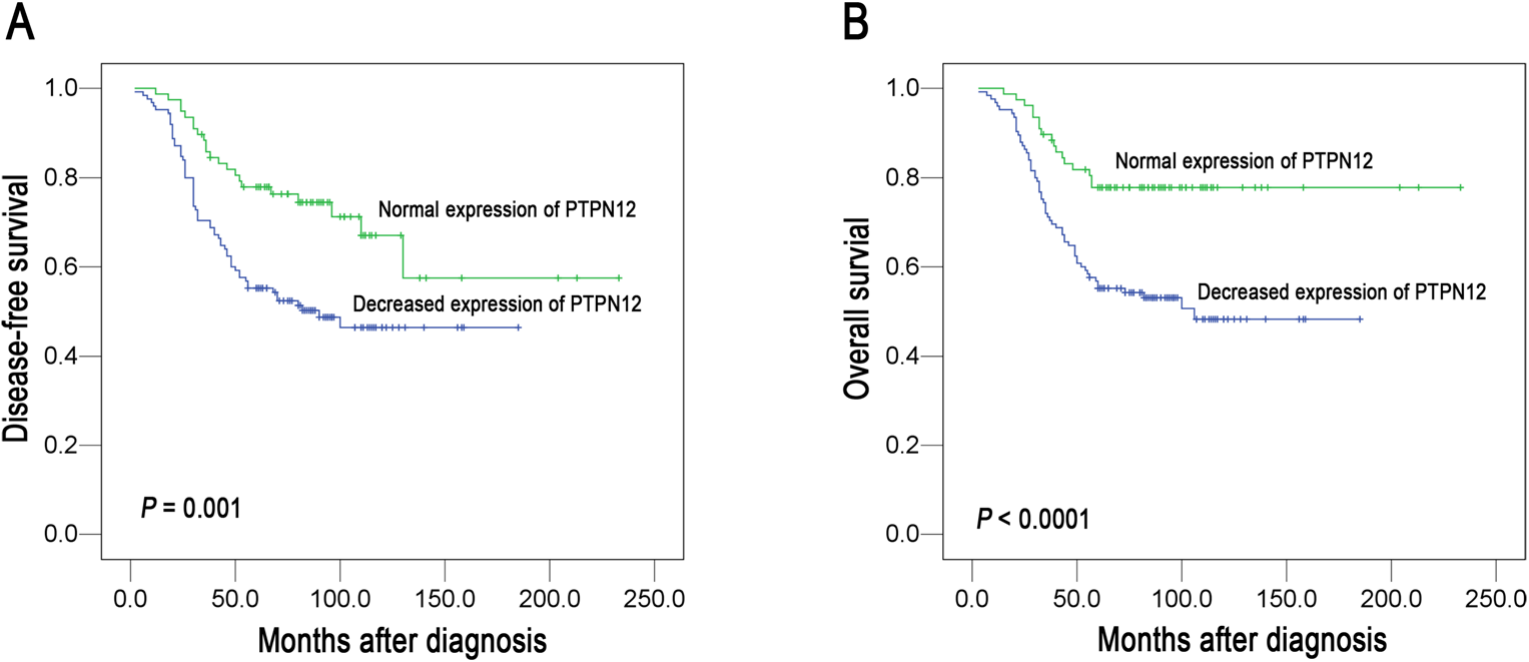
The association of PTPN12 expression with NPC patients’ survival (log-rank test). Kaplan–Meier survival analysis of PTPN12 expression for disease-free survival (**a**) and overall survival (**b**)

In this study, we firstly tested well established prognostic factors of patient survival. Univariate analysis evaluated a significant impact of well-known clinicopathological prognostic factors (i.e., T classification, N classification, distant metastasis, clinical stage) on NPC patients’ survival rates (*P* < 0.05; Table 2). Univariate analysis demonstrated that decreased expression of PTPN12 was correlated significantly with adverse disease-free survival (*P* = 0.001, Fig. 3a, Kaplan–Meier method) and overall survival (*P* < 0.0001; Table 2; Fig. 3b, Kaplan–Meier method). PTPN12 expression and other clinicopathological features were all included in multivariate analysis (Table 2). Our results showed that the decreased expression of PTPN12 was an independent prognostic factor for overall survival (Cox regression model; hazard ratio, 0.465 (95 % CI, 0.263–0.822), *P* = 0.008; Table 2).

**Table 2:** Univariate and multivariate analyses of different prognostic variables in 203 patients with nasopharyngeal carcinoma for overall survival

Variable	All cases	Univariate analysis^a^		Multivariate analysis^a^	
		Mean survival (mean ± SD)	*P* value	Hazard ratio (95 % CI)	*P* value
Gender					
Female	55	130.70 ± 12.94	0.710	0.891 (0.535–1.482)	0.656
Male	148	157.50 ± 8.14			
Age at surgery (years)					
≤45	86	121.05 ± 8.20	0.386	0.816 (0.516–1.290)	0.384
>45	117	161.22 ± 9.17			
Histological classification (WHO)					
Type II	53	179.49 ± 12.27	0.074	1.844 (1.026–3.313)	0.041
Type III	150	120.44 ± 6.29			
T classification					
T1	26	159.05 ± 15.87	0.000	0.928 (0.671–1.284)	0.651
T2	67	154.84 ± 10.66			
T3	65	178.11 ± 11.03			
T4	45	53.22 ± 4.95			
N classification					
N0	40	184.12 ± 10.87	0.000	0.986 (0.718–1.352)	0.929
N1	94	161.41 ± 10.70			
N2	48	78.22 ± 6.65			
N3	21	63.57 ± 8.17			
Distant metastasis					
0	152	171.75 ± 7.76	0.000	0.862 (0.400–1.856)	0.704
1	51	69.31 ± 6.67			
Clinical stage					
I	10	183.88 ± 18.83	0.000	2.781 (1.388–5.572)	0.004
II	55	172.81 ± 11.02			
III	78	169.91 ± 10.43			
IV	60	64.95 ± 6.06			
PTPN12 expression					
Decreased	125	110.77 ± 7.00	0.000	0.465 (0.263–0.822)	0.008
Normal	78	189.68 ± 9.29			

*CI* confidence interval, *WHO* World Health Organization

^a^ *Cox* regression model

Further analysis showed that 69 of 125 patients with decreased expression of PTPN12 and 57 of 78 patients with normal expression of PTPN12 survived more than 5 years. The 5-year overall survival rate (78 %) of patients with normal PTPN12 expression was significantly higher than that of patients with decreased PTPN12 expression (55 %), suggesting the patients with normal expression of PTPN12 had better prognosis than those with decreased expression of PTPN12.

## Discussion

NPC is characterized by poorly or undifferentiated carcinoma. It differs from nonnasopharyngeal head and neck squamous cell carcinomas in several ways, including its association with the Epstein–Barr virus (EBV) and strong sensitivity for radiotherapy and chemotherapy. High death rate is mainly due to tumor metastasis despite the new treatment that combines radiotherapy with chemotherapy [21]. Clinical staging system for NPC is now being used widely throughout the world to predict the patients’ prognosis. Patients with the same clinical staging might have different prognosis after receiving a similar treatment, therefore, it is necessary to make new objective strategies that can effectively distinguish between patients with better or worse prognosis in the same stage. Although, previous studies demonstrated many aberrantly expressed genes in NPC [22, 23]. However, novel molecular markers that can identify tumor recurrence risk remain to be urgently needed.

PTPN12 is one of the PTPs families regulating the equilibrium of tyrosine phosphorylation and plays a prominent role in tumor suppression. The PTPN12 protein is thought to act as an important regulator in controlling cell adhesion, motility, and metastasis by interacting with and inhibiting multiple oncogenic tyrosine kinases [24]. Recently, it was uncovered that PTPN12 is a tumor suppressor in human breast cancer and lung cancer, seemingly as a result of its capacity to control receptor PTK signaling [12]. In recent years, decreased expression of PTPN12 has been shown to be correlated with the development and progression of different human cancers, including breast cancer, hepatocellular carcinoma, and esophageal squamous cell carcinoma [12, 14, 17, 25]. Silencing of PTPN12 has been shown to enhance migration in ovarian cancer and colon cancer cells [13, 26]. However, expression of the PTPN12 protein in NPC and its prognostic significance in NPC are still unclear. In this study, we examined the PTPN12 protein expression in 203 NPC tissues and 40 normal nasopharyngeal mucosal tissues. Our results revealed that decreased expression of PTPN12 in normal nasopharyngeal tissues is significantly lower than that in NPC. Although the expression levels of PTPN12 in the NPC and normal nasopharyngeal tissues is significantly different, 14 of 40 normal nasopharyngeal tissues presented the decreased expression of PTPN12. The majority of NPCs had a lower expression of PTPN12 than that in nonneoplastic nasopharyngeal tissues in our study. These studies suggested that PTPN12 may be a tumor suppressor in human cancers. Further correlation analyses showed that the decreased PTPN12 expression was closely correlated with tumor stage, suggesting that PTPN12 might inhibit the differentiation and proliferation of NPC. Moreover, analysis of the association of PTPN12 expression with clinicopathological characteristics implied that the decreased expression of PTPN12 was related to lymph nodes metastasis, which was consistent with the evidence provided by Xunyi et al. [27]. Our findings support the critical role of PTPN12 as a tumor suppressor in the development and progression of NPC. Collectively, these data suggest that PTPN12 functions as a suppressor of malignant transformation and may be inactivated in human cancers. In addition, as an independent prognostic factor, decreased expression of PTPN12 was significantly correlated with the poor prognosis of NPC patients, as evidenced by univariate and multivariate Cox regression analysis. These findings were similar to that in other studies [13, 25, 26]; a strong correlation between the decreased expression PTPN12 and shortened survival was found in breast cancer, indicating that inactivated PTPN12 may result in aggressive proliferation of tumors and can be used as a key biomarker for the assessment of prognosis in human cancers.

We found that the expression of PTPN12 protein could be influenced by some factors including missense mutation of PTPN12 gene [24]. This literature reported that both human cancers and normal tissues appeared to the missense mutations of PTPN12 gene, which could produce three mutant variants of PTPN12 protein consisting of two variants with normal or high expression and a variant with decreased expression of PTPN12 in the majority of human cancer samples by immunoblotting, and the majority of normal tissues were served as nonmutant variant with normal or high expression of PTPN12, and rare tissues were regarded as mutant variant with decreased expression of PTPN12. However, we speculate that PTPN12 may maintain normal level in the NPC with normal modulation of cell-mediated immunity, once the immunity environment changed abnormal, PTPN12 might be compromised in NPC by deletion, activating silent sequence variants or loss of expression. Prior reports showed that PTPN12 acts as a negative regulator of tyrosine phosphorylation not only of p130cas and FAK as previously reported in other cells but also of TrkB [28, 29]; promoter CpG island hypermethylation occurs more frequently in breast cancer cases and breast cancer cell lines with low PTPN12 expression [27], indicating that it is a potentially mechanism leading to PTPN12 downregulation. Nevertheless, the underlying mechanism by which PTPN12 affects prognosis remains unclear and will require further investigation, Therefore, we will deeply study the mechanisms underlying PTPN12 association gene-mediated progression and metastasis of NPC in future experiments, by identifying the receptor, adapters, target proteins, and pathways of the abovementioned gene.

In summary, our study showed that the examination of PTPN12 expression by IHC could serve as an effective tool in identifying those NPC patients at increased risk of tumor invasiveness and metastasis, and also elaborate decreased PTPN12 expression as a novel adverse independent prognostic factor in NPC, which may help us find new therapeutic target.
